# Assessing the Water Quality of Lake Hawassa Ethiopia—Trophic State and Suitability for Anthropogenic Uses—Applying Common Water Quality Indices

**DOI:** 10.3390/ijerph18178904

**Published:** 2021-08-24

**Authors:** Semaria Moga Lencha, Jens Tränckner, Mihret Dananto

**Affiliations:** 1 Faculty of Agriculture and Environmental Sciences, University of Rostock, 18051 Rostock, Germany; jens.traenckner@uni-rostock.de; 2 Faculty of Biosystems and Water Resource Engineering, Institute of Technology, Hawassa University, Hawassa P.O. Box 05, Ethiopia; mihret@gmail.com

**Keywords:** water quality index, eutrophication, Lake Hawassa water quality, point sources, contaminants, monitoring and assessment

## Abstract

The rapid growth of urbanization, industrialization and poor wastewater management practices have led to an intense water quality impediment in Lake Hawassa Watershed. This study has intended to engage the different water quality indices to categorize the suitability of the water quality of Lake Hawassa Watershed for anthropogenic uses and identify the trophic state of Lake Hawassa. Analysis of physicochemical water quality parameters at selected sites and periods was conducted throughout May 2020 to January 2021 to assess the present status of the Lake Watershed. In total, 19 monitoring sites and 21 physicochemical parameters were selected and analyzed in a laboratory. The Canadian council of ministries of the environment (CCME WQI) and weighted arithmetic (WA WQI) water quality indices have been used to cluster the water quality of Lake Hawassa Watershed and the Carlson trophic state index (TSI) has been employed to identify the trophic state of Lake Hawassa. The water quality is generally categorized as unsuitable for drinking, aquatic life and recreational purposes and it is excellent to unsuitable for irrigation depending on the sampling location and the applied indices. Specifically, in WA WQI, rivers were excellent for agricultural uses and Lake Hawassa was good for agricultural uses. However, the CCME WQI findings showed rivers were good for irrigation but lake Hawassa was marginal for agricultural use. Point sources were impaired for all envisioned purposes. The overall category of Lake Hawassa falls under a eutrophic state since the average TSI was 65.4 and the lake is phosphorous-deficient, having TN:TP of 31.1. The monitored point sources indicate that the city of Hawassa and its numerous industrial discharges are key polluters, requiring a fast and consequent set-up of an efficient wastewater infrastructure, accompanied by a rigorous monitoring of large point sources (e.g., industry, hospitals and hotels). In spite of the various efforts, the recovery of Lake Hawassa may take a long time as it is hydrologically closed. Therefore, to ensure safe drinking water supply, a central supply system according to World Health organization (WHO) standards also for the fringe inhabitants still using lake water is imperative. Introducing riparian buffer zones of vegetation and grasses can support the direct pollution alleviation measures and is helpful to reduce the dispersed pollution coming from the population using latrines. Additionally, integrating aeration systems like pumping atmospheric air into the bottom of the lake using solar energy panels or diffusers are effective mitigation measures that will improve the water quality of the lake. In parallel, the implementation and efficiency control of measures requires coordinated environmental monitoring with dedicated development targets.

## 1. Introduction

Surface waters play the lion’s share in transportation and assimilation of municipal and industrial effluents and agricultural runoff; consequently, they are most prone to pollutants [1]. Industrialization on top of rapid population growth triggers land development along a river basin, exerting greater pressure on water bodies by giving rise to water pollution and ecological impediment [2].

Surface water pollution with chemical, physical and biological contaminants by anthropogenic activities from the point and non-point sources is of great environmental consideration all over the world [3].

In Ethiopia, due to lack of access to improved water supply and sanitation, people are suffering from water communicable diseases that are associated with unsafe and inadequate water supply. Additionally, water quality problems are booming in water sources of the country that demand effective monitoring and evaluation for the proper protection of water sources from contamination [4]. A study conducted by Angello et al. [5] revealed that increased urbanization has prompted the opening of medium- to large-scale industries resulting in pollution of most surface water resources by the wastewater released from different sources. Wastewater from residential areas, runoff from urban and agricultural activities near surface waters contribute a significant quantity of contaminants. Additionally, industrial effluents that are released directly with little or no treatment into surface water bodies were one of the major pollution sources in Akaki river. Lake Hawassa is one of the major Ethiopian Rift Valley Lakes basins and it is used for manifold purposes like irrigation, human consumption by some city and rural inhabitants close to the city, recreation, livestock, watering and fish farming [6].

Studies showed a high amount of pesticides in water, sediments and fish species in Lake Hawassa due to its exposure to effluents from factories, urban and agricultural runoff. As a result, the lake is contaminated and affects the biodiversity of the aquatic ecosystem including fish [7]. The growth and death of floating aquatic plants are supplementing the algal growth and sediments that accumulates at the bottom of the lake and yield cultural eutrophication [8].

The impact on the lake is mainly due to anthropogenic activities in its catchment. Sanitation is a great concern. Most of the population, even in the inner part of the city of Hawassa are using latrines. Larger buildings provide conventional flushing systems but without any wastewater treatment. Furthermore, industrial and commercial pollution sources (i.e., BGI, Moha soft drinks, flour factory and ceramic factory) are known to release effluents into streams or rivers that end up in the small marshy land after which Tikur-Wuha river got its name and fed Lake Hawassa. In addition, Hawassa Industrial park and the Referral hospital are releasing their effluents directly to the lake. This is a danger to the people that depend on rivers, streams and lake for domestic and other uses and to the existence of marine species [9].

The study conducted by Zemede et al. [10] made use of different water quality indices and discovered that the status of water quality of Lake Hawassa was under the hypertrophic condition and generally unsuitable for all uses.

Evaluating the status of water quality from analytically determined data of parameters with the international and national permissible values does not guarantee the whole visualization of the water quality situation. Therefore, developing a sole value of WQI that can convey information more easily in a way that can be more rapidly understood than a list of large parameter values is vital [11].

The water quality index (WQI) is a very effective tool to integrate and deliver information regarding water quality to experts and the wider community [12] and is also used to associate the water quality of different sources and monitoring sites [13]. By addressing usage criteria, the negative impact of environmental pollution becomes tangible. It is a unit-less number that combines information from manifold analytical data into a sole aggregate through a method that portrays the situation of water quality well for the public and experts [14].

Numerous indices had been established so far in various parts of the world to estimate water quality status and pollution extents of the water bodies. Just to mention a few, the National Sanitation Foundation (NSF) index Water quality index (NSF WQI) [15], Canadian Council of Ministries of the Environment Water Quality Index (CCME WQI) [16], Oregon Water quality index (Oregon WQI) [13], Bascarón index [17], Fuzzy index [18], Boyacioglu’s index [19], Weighted Arithmetic water quality index (WA WQI) [20] and many more. NSF WQI, CCME WQI, Oregon WQI and WA WQI are the most widely used techniques around the globe [20].

To conduct all-inclusive water quality valuation for lakes besides the water quality indices approach, implementing the trophic state index approach to identify the productivity of the lake is mandatory. The Organization for Economic Cooperation and Development provides specific criteria for temperate lakes in terms of the average annual values of total phosphorus, chlorophyll a and Secchi depth [21]. The limitations of these criteria were that the same lake could be assigned in one or another trophic class based on the applied parameters. Studies showed the computation of trophic state ranking of lakes or reservoirs from variables like Total nitrogen (TN), Total phosphorous (TP) and phytoplankton mass that are responsible for eutrophication of lakes. Kratzer and Brezonik [22] established an index for eutrophication based on TN from the Carlson index; whereas Boyle et al. [23] established a pH and dissolved oxygen-based index. Additionally, Hailin and Baoyin [24] also formulated an index that depends on Biochemical demand (BOD) by formulating statistical association between chlorophyll a (Chl-a), TP and TN. Köklüa and Alkış [25] also established a new trophic level index using quality indicators that are known by affecting eutrophication with limited applicability. The Carlson trophic status index (TSI) has long been established to evaluate the trophic state of lots of reservoirs and lakes and is determined using the procedures explained by Carlson [26]. Carlson trophic status index TSI has been commonly used approach-and separately estimated from total nitrogen concentration, Secchi depth (SD), (chl-a) and total phosphorus concentration (TP) [27].

This study has, therefore, tried to elucidate the use of weighted average (WA), CCME and TSI water quality indices to categorize the water quality of Lake Hawassa Watershed and identify the trophic state of Lake Hawassa.

## 2. Materials and Methods

### 2.1. Study Area

**Figure 1:**
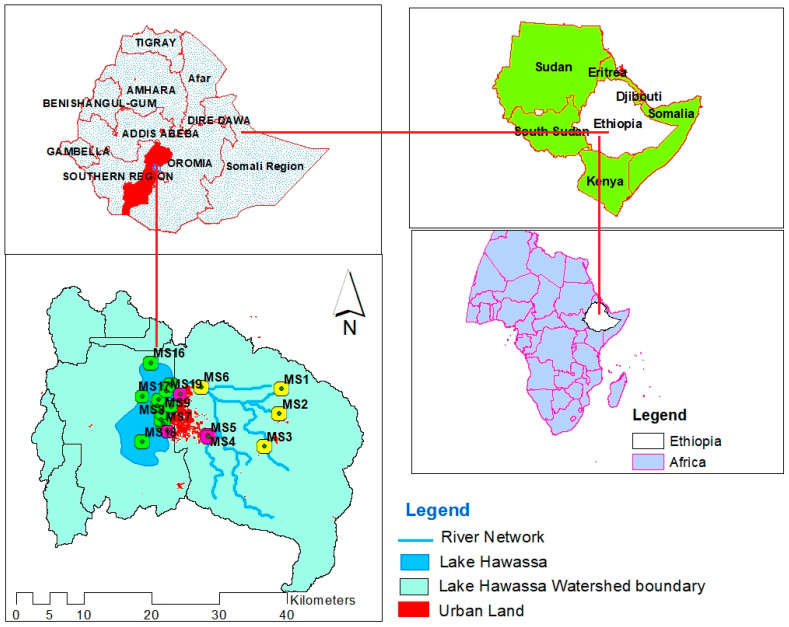
Location of Lake Hawassa Watershed and monitoring stations.

Lake Hawassa watershed is located in the center of the Rift Valley Lakes basin, between latitudes of 6°4′45″ N to 7°14′49″ N and longitudes of 38°16′34″ E to 38°43′26″ E [28,29]. Amongst the seven lakes in the Rift Valley Lakes basin, Lake Hawassa is located between the latitude of 6°33′–7°33′ N and longitude of 38°22′–38°29′ E (Figure 1). The Lake Hawassa watershed is located in Oromiya and Sidama regional state, having a total area of 1407 km^2^ and 113 km^2^ of which is Lake surface area [30]. Streams from the eastern catchment flow to Lake Cheleleka and are drained by the Tikur-Wuha river that feeds the Lake Hawassa. This river water has been extensively affected by various point sources [31]. The lake has no surface water outflow except evaporation and abstraction and it is used for commercial fishing and tourist destinations [32].

The months from April to October are wet and humid; the main rainy season is between July and September, having mean annual precipitation of about 955 mm. The mean minimum precipitation is 17.8 mm in December (dry season) and the mean maximum precipitation is 119.8 mm in August (rainy season) [33]. The long-term mean annual temperature is around 19 °C while the mean monthly evapotranspiration in the low lands ranges from 39 mm in July to 100 mm in January [34].

### 2.2. Sampling and Analysis of Monitoring Parameters

Water and effluent samples were collected from rivers, point sources and different monitoring points from Lake Hawassa Watershed depending on the Lakes exposure to anthropogenic activities. The coordinate of each sampling station was determined applying GNSS.

In total 19 monitoring sites were selected purposively in close proximity to potential pollutants, accessibility, availability of point and non-point sources and level of disturbance where their effluents end up in the lake.

Four (4) monitoring sites selected from the eastern catchment of Lake Hawassa Watershed that exclusively comprises rivers namely Wesha (MS1), Hallow (MS2), Wedessa (MS3) and Tikur-Wuha (MS6) river mouths of the respective sub-watersheds. Eleven (11) monitoring sites were evenly distributed along the entire course of Lake Hawassa for estimation of the eutrophic status of the lake and water quality monitoring. Three (3) monitoring sites were selected from the industrial disposal site and one monitoring site is from the health care center as shown in Table 1 and Figure 1.

Samples of lakes and rivers were collected from different depths and intervals of the entire water column and mixed to make the sample composite. Referral hospital, Hawassa Industrial park, St. George Brewery industry (BGI) and Moha soft drinks factory effluents were collected from their respective oxidation ponds and discharge points using pre-cleaned 2 L polyethylene plastic bottles sterilized for Biochemical oxygen demand (BOD_5_) and Chemical oxygen demand (COD). The physicochemical and biological properties of water quality parameters can be monitored based on the required water parameters of concern. BOD and COD were selected to assess the presence of organic pollution. TN, TP, Nitrate (NO_3_^)^ and Soluble reactive phosphorous (SRP) were selected to monitor non-point sources pollution from agricultural land, urban drainage and residential lawns and the use of inorganic nitrogen fertilizers. Magnesium ion (Mg^+2^), Calcium ion (Ca^+2^), Sodium ion (Na^+^), Potassium ion (K^+^) and their empirical values Sodium Adsorption Ratio (SAR), Kelly’s ratio (KR), Magnesium Adsorption ratio (MAR) and Soluble sodium percentage (SSP) were selected to test the suitability of water for agricultural use and Mg^+2^, Ca^+2^, Na^+^, K^+^ were also selected to monitor water suitability for drinking purposes. Nitrite (NO_2_^−^) and Ammonia (NH_3_) were selected to monitor the toxic effect of water for human consumption and marine life. Recreational water suitability is based on turbidity, Secchi depth (SD), Dissolved oxygen (DO) and BOD. TN, TP, Secchi depth and chlorophyll a (chl-a) were selected to monitor the trophic state of lake Hawassa. Turbidity was selected to measure the presence of suspended material whereas EC and TDS were used to monitor the amount of total dissolved substances in water or effluent. pH was selected to survey acidity or alkalinity of water or effluent and the temperature was selected as it is correlated negatively or positively with most of the water quality parameters. All the parameters analyzed in Table 2 below were generally selected by taking into consideration the appropriateness of water for human consumption, agricultural use, marine life and recreational uses.

Water sample collection, handling, preservation and treatment techniques followed the standard methods outlined for the examination of water and wastewater by the American public health association guidelines [35].

#### Un-Ionized Ammonia Determination from Total Ammonium Nitrogen (TAN)

The mass action law in its logarithmic form (1) calculated the un-ionized free ammonia. The pKa as function of temperature was taken from [36]:

(1) $$\%\;\mathrm{Un}-\mathrm{ionized}\;{\mathrm{NH}}_{3}-N=\frac{1}{\left(1+10^{\left({\mathrm{pK}}_{a}-\mathrm{pH}\right)}\right)}$$

(2) $${\mathrm{pK}}_{a}=\frac{0.09108+2729.92}{\left(T_{k}\right)}$$

where, T_k_ is temperature in kelvin (273 + °C).

### 2.3. Weighted Arithmetic Water Quality Index Method (WA WQI)

In the literature, the weighted arithmetic water quality index method (WA WQI) was developed [10,20,37,38,39,40] in a large number of studies.

WQI was determined by utilizing the weighted arithmetic index method in the following steps. Water quality parameters (n) and quality rating ($q_{n}$) associated to the nth parameter is a number defining the relative value of this parameter in the polluted water with respect to its standard value.

#### Methodology in Calculating WQI Using the WA WQI Method

WQI initially proposed by [41] and advanced by Brown et al. [42] as cited by [20,43,44].

Calculate unit weight ($W_{n}$) for the nth parameters:

(3) $$W_{n}=\frac{K}{S_{n}}$$

Define proportionality constant “K” value using formula:

(4) $$K=\frac{1}{\sum_{i=1}^{n}\frac{1}{S_{n}}}$$

Sub-index or quality rating ($q_{n}$) for nth parameter can be calculated using the following formula:

(5) $$q_{n}=100\;*\left(\frac{V_{n}-V_{i}}{V_{s}-V_{i}}\right)$$

where, $v_{s}$ is Standard value for the nth parameter, $v_{n}$ is measured value of the nth parameter, $v_{i}$ is the ideal value of nth parameter and in most cases $v_{i}=0$ except for pH (7) and DO (14.6) [45].

Quality rating ($q_{n}$) for pH and DO can be determined using the formula given below.

(6) $$q_{\mathrm{pH}}=100*\left(\frac{V_{\mathrm{pH}}-7}{V_{s}-7}\right)$$

(7) $$q_{\mathrm{DO}}=100*\left(\frac{V_{\mathrm{DO}}-14.6}{V_{s}-14.6}\right)$$

The water quality index (WQI) determined using the formula below and the water quality rating [46] depicted in Table 3.

(8) $$\mathrm{WQI}=\frac{\sum_{i=1}^{n}q_{n}*W_{n}}{\sum_{i=1}^{n}W_{n}}$$

### 2.4. Canadian Council of Ministries of the Environment Water Quality Index (CCME WQI)

In CCME WQI the WQI can easily be adopted to the local situations as it permits flexibility in selecting parameters. A number of studies applied CCME WQI in different parts of the world for the evaluation of suitability of water quality for drinking, irrigation and aquatic life [47] in Turkey [48], India [12,49,50], Albania [51] and Iran [52,53] and in different parts of Ethiopia [10,54,55] and elsewhere.

In CCME WQI, three factors, Scope (F1); Frequency (F2) and Amplitude (F3) are integrated mathematically from designated water quality objectives [52].

They provide an arithmetic value of CCME WQI water quality status in between 0 (poor) and 100 (excellent) in five descriptive classes as described in Table 4 [16,48,56,57].

#### CCME WQI Calculation Methods

The WQI was computed based on the three parameters F1, F2 and F3 for the intended purposes.

F_1_ (Scope) represents the number of water quality variables that violate the standards:

(9) $$F_{1}=\left(\frac{Number\;of\;failed\;variables}{Total\;number\;of\;variables}\right)*100$$

F_2_ (Frequency) represents the number of times the standards are violated:

(10) $$F_{2}=\left(\frac{Number\;of\;failed\;tests}{Total\;number\;of\;tests}\right)*100$$

F_3_ (Amplitude) represents the amount by which the standards are not met and determined in three steps.

The number of times by which an individual concentration is greater than (or less than, when the objective is a minimum) is termed excursion and expressed as follows:

When the test value must not exceed the objective,

(11) $${\mathrm{Excursion}}_{i}=\left(\frac{\mathrm{Failed}\;\mathrm{test}\;\mathrm{value}}{\;\mathrm{Objective}\;j}\;\right)-1$$

When the test value must not fall below the objective,

(12) $${\mathrm{Excursion}}_{i}=\left(\frac{\mathrm{Objective}\;j}{\mathrm{Failed}\;\mathrm{test}\;\mathrm{value}}\;\right)-1$$

(13) $$\mathrm{nse}=\sum_{i=1}^{n}\frac{\mathrm{Excursion}\;j}{\mathrm{Total}\;\mathrm{number}\;\mathrm{of}\;\mathrm{tests}}$$

F_3_ is then determined by an asymptotic function that scales the normalized sum of the excursions from objectives (nse) to yield a range between 0 and 100:

(14) $$F_{3}=\left(\frac{\mathrm{nse}}{0.01\mathrm{nse}+0.01}\right)$$

Finally, CCME WQI:

(15) $$\mathrm{CCMEWQI}=100-\left(\frac{\sqrt{F_{1}{}^{2}+F_{2}{}^{2}+F_{3}{}^{2}}}{1.732}\right)$$

### 2.5. Evaluation of the Trophic Status Using Carlson Trophic State Index (TSI) Model

The Carlson Trophic State Index is the conventional approach that depends on the changes in nutrient level of lakes and reservoirs that are responsible for algal biomass production and that were known by decreasing Secchi disk transparency [26]. The Carlson’s Trophic State Index is the most widely used scheme [27]. It integrates all the parameters into a single form so that a general condition could easily be communicated [22,26,27].

#### Method to Determine Trophic State Index

Various approaches have been established to quantify the trophic state (TS) of lakes. Carlson’s Trophic Status Index was selected for the present study were given below in Table 5 [58]. Carlson’s TSI is a common technique to distinguish a lake’s trophic state and presented Range of the Carlson’s Trophic Status Index (TSI) values and classification of lakes [27]. This method delivers a more detailed calculation of the trophic status than the other conservative approaches that only provide a coarse trophic state estimation presented in [22,59,60] 

The following equations can be used to compute the Carlson’s TSI.

(16) TSI (TN)=54.45+14.43∗ln (TN) (mg/L)

(17) TSI (TP)=14.42∗ln (TP)+4.15 (μg/L)

(18) TSI (Chl a)=9.81∗ln (chl a)+30.6 (μg/L)

(19) TSI (SD)=60−14.41∗ln (SD)(m)

where SD the Secchi depth, chla is chlorophyll a, TP is total phosphorous and TN is total nitrogen

Eutrophic ecosystems are described by referring to the supplies of growth-limiting nutrients and water having relatively large supplies of nutrients, and are termed eutrophic (well nourished), poor nutrient supplies (oligotrophic) and intermediate nutrient supplies are termed mesotrophic They categorize the trophic status of the lake based on the total nitrogen (TN) and total phosphorous (TP) loads in Table 6 that are supposed to be accumulated in the lake bottom [61,62,63].

## 3. Result and Discussion

### 3.1. Water Quality Status for Envisioned Purposes

Selection of parameters is imperative for calculation of WQI and depends on the intended use. A selection of large number of parameters broaden the water quality index, pH, EC, TDS, turbidity, NH_3_, NH_3_-N, NO_2_-, NO_3_^−^, NO_3_-N, DO, BOD, COD, Mg^+2^, Ca^+2^, Na^+^, K^+^, temperature, SAR, KR, MAR, SSP and SD are used to evaluate the suitability of Lake Hawassa Watershed for drinking, irrigation uses, recreation and aquatic life [46].

#### 3.1.1. pH

**Figure 2:**
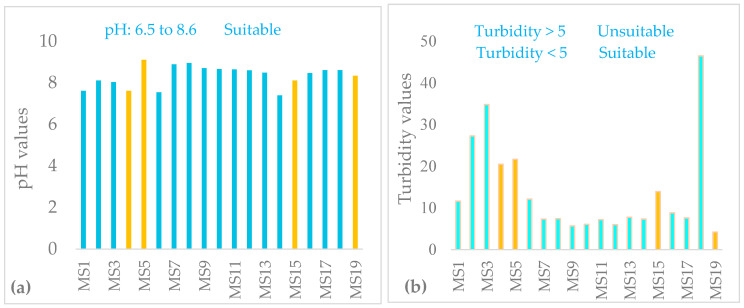
pH (**a**) and turbidity (NTU) (**b**) in the water and wastewater samples (n = 19) collected over 19 monitoring stations with wastewater samples labeled yellow in the Lake Hawassa Watershed.

In WQI computation pH is an imperative parameter that determines the suitability of water for the various purposes. The results of the study depicted in Table 7 and Figure 2a. The pH value of the water indicated that the watershed is slightly alkaline as it varied between 7.6 (MS1) and 9.1 (MS5). However, the pH of the Lake Hawassa Watershed is within the permissible limits i.e., 6.5–8.5/9 [64,65,66] for rivers and lakes. A high value of pH is observed in the wet season, which might be due to the dissolution of carbon dioxide and nutrients produced during bacterial decomposition of domestic wastes near the lake [67].

The average pH values for the upper and middle monitoring stations of four rivers is 7.99 having an average value of 7.6 at (MS1), 8.1 at (MS2), 8.03 at (MS3) and 7.55 at (MS6) all of which are in accordance with the permissible limit prescribed by the WHO. The finding of this study is comparable with the previous studies conducted by Kebede et al. [30] and Teshome [55] on the eastern catchment of Lake Hawassa Watershed.

The average value of pH measured from point sources in monitoring stations MS4, MS5, MS15 and MS19 were 7.62, 9.1, 8.1 and 8.34, respectively. The average pH value of the Lake Hawassa is 8.5 for this study and comparative observations were made in Lake Hawassa with previous studies conducted by Abiye [33] who found an average value (pH = 8.5) and showed an increment from the results of Worako [6] and Yogendra and Puttaiah [44] (pH = 7.5) elsewhere. It is very probable that the increased pH values are mainly due to the consumption of dissolved carbon dioxide by the autotrophic biomass in the upper layer of the eutrophic lake. These conditions may completely change in deeper layers, where due to the absence of light heterotrophic degradation processes should be dominant [68].

#### 3.1.2. Turbidity

The turbidity in monitoring stations ranges from 4.24 to 46.5 NTU. The average turbidity value for rivers were 21.5 NTU, Lake Hawassa was 10.7 NTU and point sources were 15.1 NTU. The turbidity of the study watershed is higher than the recommend value by [16,65,69] for drinking and aquatic life, except at MS19 (4.24). The highest value of turbidity was recorded at MS18 (46.5 NTU) followed by MS3 (34.8) sampling stations; whereas, the minimum value of turbidity was recorded at MS19 (4.24 NTU) sampling station (Table 7 and Figure 2b).

The high values of turbidity could be attributed to agricultural and urban runoff from the catchment area, the loading of rivers and the lake with silt during the wet season and high human intervention in the river and lake water for multi purposes, and discharge of effluents from MS4, MS5, MS15 and MS19. The high turbidity value from industries might be due to organic matter decomposition present in the effluents [9,10,67,70]. There is also a moderate positive correlations observed between turbidity with chl-a (r = 0.6 at *p* < 0.005) and COD values (r = 0.6 at *p* < 0.005). The result of this study also showed Lake Hawassa water clarity is low as evidenced by lower SD (0.76 m) and TSI of 65.4 leading to high nutrient concentrations, high algal blooms but low light penetration and low water clarity. Lack of clarity limits the light penetration rendering greater impacts on algae and macrophytes while degradation of organic matter in deeper layers can lead to the depletion of oxygen and subsequently fish kill [71]. The recreational use of water is reduced due to lack of clarity as the value of Secchi depth for lake Hawassa was lower than the recommended limit of 1.2 m [72] and turbidity value was higher [73]. Most natural waters have turbidities less than 50 NTU [74].

High turbidity also reduces the efficiency of disinfectant in water supplies for drinking purposes and cause a health risk by enhancing the growth of bacteria during storage. Hence, special attention ought to be given to the turbidity of Lake Hawassa Watershed as its value lies within a level that could pose a health risk and reduces the disinfection process in water supplies.

#### 3.1.3. Nitrate (NO_3_^−^), Nitrate-Nitrogen (NO_3_-N) and Nitrite (NO_2_^−^), Nitrite Nitrogen (NO_2_-N)

**Figure 3:**
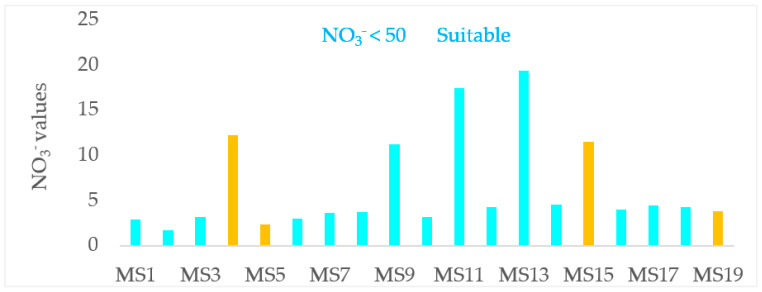
Nitrate (NO_3_^−^) concentrations (mg/L) in the water and wastewater samples (n = 19) collected over 19 monitoring stations with wastewater samples labeled yellow at the Lake Hawassa Watershed.

The WHO guideline recommends 50 mg/L for nitrate ion, (11 mg/L) as NO_3_-N and 3 mg/L of nitrite ion and (0.9 mg/L) as NO_2_-N for safe human consumption. In the studied watershed, these values were far below the prescribed limit. The average nitrate (NO_3_^−^), nitrate-nitrogen (NO_3_-N) and nitrite (NO_2_^−^) concentrations of rivers were 2.7, 0.6 and 0.06 mg/L respectively and that of point sources were 7.5, 1.7 and 0.06 mg/L, respectively. The average nitrate (NO_3_^−^), nitrate-nitrogen (NO_3_-N) and nitrite (NO_2_^−^) concentrations of Lake Hawassa were 7, 1.7 and 0.04 mg/L, respectively (Table 7 and Figure 3). The study conducted by Camargo and Alonso [75] have shown that a NO_3_-N concentration of 10 mg/L NO_3_-N can adversely affect sensitive aquatic animals in the course of long-term exposure.

The measured concentrations are significantly higher than the study conducted by Tilahun and Ahlgren [29] on Hawassa and Chamo lakes and reported that the mean concentration of NO_3_-N was about 0.0025 and 0.003 mg/L in Lakes Hawassa and Chamo respectively. This indicates a dramatic worsening of the situation in Lake Hawassa in the last 10 years. This might be due to input of fertilizer application by agricultural land, effluents from industrial facilities and sewage from health care centers and domestic sewage from service rendering facilities and urban run off as compared to the last decade. Similarly, Tibebe et al. [76] and Fetahi [77] reported lower average results of NO_3_-N (0.21) and (0.042) mg/L in Lakes Zeway and Hayq. Currently, the people in the peripheries of the city uses the lake for drinking purpose as well.

##### Nitrite-Nitrogen (NO_2_-N)

The average nitrite-nitrogen (NO_2_-N) concentrations of rivers, point sources and Lake Hawassa were 0.02, 0.01 and 0.02 mg/L respectively (Table 7). Nitrite in excess concentration is toxic to fish and aquatic species [75]. The mean NO_2_-N concentration observed in this investigation (0.01 mg/L) for Lake Hawassa was comparable to that of Tamire and Mengistou [78] who reported 0.01 mg/L and lower than Tibebe et al. [76] who reported 0.5 mg/L for Lake Ziway, respectively.

#### 3.1.4. Dissolved Oxygen (DO)

The current investigation showed the variation in the DO value of Lake Hawassa Watershed ranged from 3.12 to 5.2 mg/L and the highest value was recorded at MS1 (5.4) Wesha river in the upper catchment and the lowest value was recorded at MS5 (0.9) at Moha soft drinks factory factory). The average DO value of rivers were 5 mg/L, Lake Hawassa was 4.3 mg/L and point sources were 2.2 mg/L (Table 7).

The DO levels were below the acceptable limit (<5 mg/L) of EPA for samples collected from lakes, indicating the impairment of the water body for aquatic life [79]. The major cause for lowering of DO was the point sources having the average DO value of 2.2 mg/L. The findings of this study agree with the previous studies conducted by Abiye [33], Zemede et al. [10] and are much lower than that of Worako [6] on Lake Hawassa.

The amount of DO regulates how the species of phytoplankton and zooplankton are distributed in aquatic ecosystems [44]. Decomposition of nutrient and submerged plants on the lake, biodegradable organic matter and urban and agricultural runoff might be the reason for the presence of low dissolved oxygen [80,81]. Most of the species of fish can survive short-term exposure to the lowered DO [82] and the threshold of 3 mg/L dissolved oxygen level should be maintained to safeguard from significant critical effects [83].

#### 3.1.5. Chemical Oxygen Demand (COD) and Biological Oxygen Demand (BOD_5_)

##### Chemical Oxygen Demand (COD)

**Figure 4:**
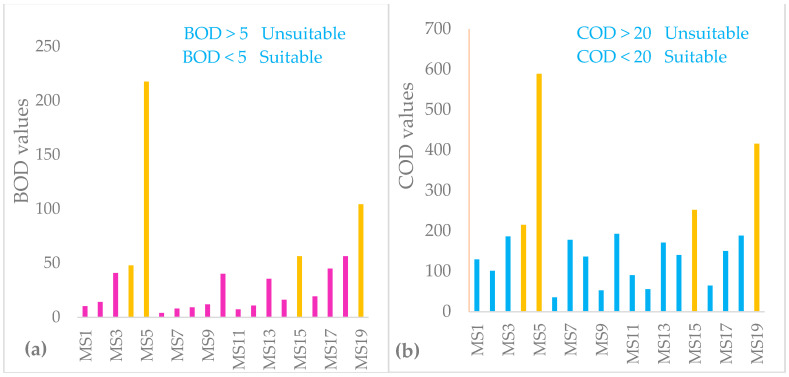
Biological oxygen demand (BOD) (**a**) and chemical oxygen demand (COD) (**b**) concentrations (mg/L) in the water and wastewater samples (n = 19) collected over 19 monitoring stations with wastewater samples labeled yellow at the Lake Hawassa Watershed.

COD represents the total oxygen demand of the organic matter, independent from its origin and degradability. The average COD value of rivers were 113 mg/L, Lake Hawassa was 129 mg/L and point sources had an average value of 368 mg/L (Table 7 and Figure 4b). COD of the industrial point sources can be clearly assigned to primary pollution while a COD of the lake water may be partly caused by the phytoplankton.

The COD analysis for Lake Hawassa ranges from 52 mg/L (MS9) to 193 mg/L (MS10) and higher values of COD were observed at sampling locations of MS7 (178 mg/L), MS8 (136 mg/L), MS10 (193 mg/L), MS13 (171 mg/L), MS14 (140 mg/L), MS17 (150 mg/L) and MS18 (188 mg/L). The most heavily polluted site was MS10 (Fikerhayk recreation center), where there are hotels, restaurants, cafés, boating activities and it is that part where the lake experiences maximum human intervention almost every day whereby flushing and forgetting is business as usual. MS7 (Amora-Gedel) and MS8 (Gudumale) was heavily polluted sites where there was a small fish market around that site. Additionally, the Gudumale recreation center situated near this site was serving as a location for marriage ceremonies, for various events like gatherings, and the people of Sidama celebrate a new year (Fiche-Chambala) at this site. All the waste products were discharged from the two sites to the nearby lake in diffused form and also people around these site uses the lake water for cloth washing and bathing purposes. MS17 and MS18 monitoring sites were located on the western (north west to south west) sides of the lake. Although there is no point source pollution in these parts of the lake but there are enormous anthropogenic activities in the form of nonpoint source of pollution from the recreational activities, agricultural runoff and animal waste. MS13 monitoring stations located near Haile resort and Hawassa industrial park where the park directly discharges its effluent directly to the lake. MS14 was also located on the northeast part of the lake where Tikur-Wuha river joins the lake at this station and there are recreational and fishing activities. Moreover, the urban runoff makes its way to the lake from the above monitoring stations during the rainy season. Generally, the COD value were higher in monitoring stations indicating the presence of higher organic matter impeding the lake water quality. Hence, designing and implementing riparian buffers strips of vegetation and grasses around the periphery of lake Hawassa is imperative to safeguard it. The findings are higher than that of Abiye [33] who found an average value of 78 mg/L for lake Hawassa owing to the impact of urbanization-related activities, such-as domestic sewage and urban runoff that contains organic matter. This reveals that there has been a visible change of water quality impediment in the last 13 years due to the domestic and industrial sewage and urban runoff.

##### Biological Oxygen Demand (BOD_5_)

The average BOD_5_ value of rivers were 17.3 mg/L, Lake Hawassa was 23.6 mg/L and point sources were 106.6 mg/L showing point sources are the cause for pollution of the lake (Table 7 and Figure 4a). Rivers or lakes are considered unpolluted if the average value of BOD < 3 mg/L, however, BOD > 5.0 mg/L was recorded in all 19 monitoring stations signposting possible pollution [38].

Releasing liquid wastewater with higher BOD causes impairments in water quality such as DO decline and fish kills in the receiving water bodies [84]. The concentration of BOD5 in the area under investigation is beyond the permissible limits of WHO and EPA guidelines (<5 mg/L) for human consumption and aquatic life in the study watershed; which indicates the water in the watershed is highly polluted by organic matter.

BOD is a parameter used to judge the presence of organic load in a water body and also used as an indicator of whether a water body is in a eutrophied state. Higher BOD levels of a water body are associated with lower dissolved oxygen levels [44]. Those involved in recreational facilities are probably most at risk due to eutrophic conditions [85]. The findings are lower than that of Zemede et al. [10] who recorded an average value of 44.9 mg/L for Lake Hawassa.

#### 3.1.6. Total Ammonia Nitrogen (NH_4_-N + NH_3_-N)

Total ammonia nitrogen (TAN) is the sum of ammonium nitrogen in ionized form (NH_4_-N) and un-ionized ammonia nitrogen (NH_3_-N) that are the principal water quality indicators, with their relative concentrations dependent on both pH and temperature. The un-ionized form is toxic as it is neutral and can penetrate gill membranes more readily than the NH_4_^+^ ions. Studies showed the toxicity of total ammonia ascribed due to the effect of free ammonia only [86,87].

Aquatic organisms are extremely sensitive to elevated levels from the natural ammonia level and the un-ionized form of ammonia is deadly to aquatic animals including fish. At the pH of 8.75 to 9.75, unionized ammonia and ammonium ions coexists in aqueous state and the fraction of un-ionized ammonia increases with temperature and pH. When the pH (<8.75), ammonium ions are the principal species in water bodies, unionized ammonia becomes the pre-dominant species at pH (>8.75) [88,89,90]. In the lake watershed under investigation, the mean ammonium nitrogen ranges from 0.12 mg/L (MS19) to 16.8 mg/L (MS15). The average ammonium nitrogen value of rivers was 1.17 mg/L, Lake Hawassa was 2.35 mg/L, and point sources were 7.2 mg/L (Table 7). The findings revealed the point sources were the major source for ammonium nitrogen to the rivers and Lake Hawassa. While ammonium is less toxic and the most desirable source for phytoplankton growth, it becomes toxic to fishes and may result in eutrophication of lakes at higher concentrations [91,92].

On the other hand, a good quality water body must have an ammonia levels less than 0.05 mg/L and when this level goes beyond 2 mg/L fish are killed [75,93,94]. Nonetheless, in the watershed under investigation, the mean ammonia ranges from 0.01 mg/L (MS6) to 8.9 mg/L (MS5). The average ammonia value of rivers was 0.11 mg/L, Lake Hawassa was 0.94 mg/L, point sources were 2.9 mg/L, and these values are higher than the recommended value (Table 7). In addition, point sources were contributing larger amounts of ammonia to the river and Lake Hawassa. The findings are in line with the previous studies conducted by Kebede et al. [30] and Teshome [55] on the eastern catchment of Lake Hawassa. Ammonia is an indicator for elevated pollution from organic substances producing a noxious odors and are often indicative of sewage pollution and agricultural runoff [51,89].

#### 3.1.7. Soluble Reactive Phosphorus (SRP)

The mean values of SRP ranged from 1.8 to 76.8 mg/L and higher values observed at MS5 (Moha soft drinks factory). The recommended concentration of phosphate for good quality water that maintains the aquatic life is in the range of 0.005 to 0.02 mg/L [69].

The average SPR concentration in the upper catchment of three rivers (MS1, MS2 and MS3) was 6.56 mg/L which is higher than the study conducted by Kebede et al. [30]. Moreover, the SPR of the four rivers including Tikur-Wuha river (MS1, MS2, MS3 and MS6) was 6.5 mg/L (Table 7), that is greater than the study conducted on the eastern catchment by Teshome [55]. This might be due to increased population due to urbanization, use of detergents, the practice of open defecation, and intensive usage of fertilizers in agricultural land.

The overall mean of SRP concentration in Lake Hawassa was 3.34 mg/L that is greater than the previously reported value of Zinabu et al. [95], Tilahun and Ahlgren [29] and Tamire and Mengistou [78] which was 0.035, 0.01 and 0.029 mg/L respectively. Hence, the phosphate level in the study watershed exhibited non-conformity with the standard values that can exacerbate eutrophication in fresh water systems and loss of aquatic biodiversity. This might be due to an increased usage of fertilizers in agricultural lands, industrial effluents, excessive usage of detergents in domestic and industrial facilities, soil erosion, and increased sewage pollution showing an ongoing pollution of the lake. Additionally, phosphate carrying pollutants like fertilizers, domestic wastewater, detergents, industrial effluents, runoff from agricultural and urban setup leading to algal blooms, eutrophication and elevated BOD [85,96]. The results of this study showed an increment from the previous studies conducted on the eastern catchment of Lake Hawassa by Kebede et al. [30], Teshome [55] and on Lake Hawassa conducted by Worako [6].

### 3.2. Summary of Irrigation Indices

#### 3.2.1. Total Dissolved Solids (TDS) and Electrical Conductivity (EC)

**Figure 5:**
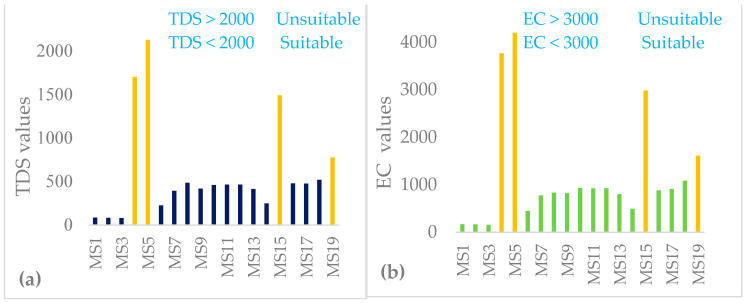
Total dissolved solids (TDS) (**a**) concentrations (mg/L) and electrical conductivity (EC) (**b**) concentrations (µS/cm) in the water and wastewater samples (n = 19) collected over 19 monitoring stations with wastewater samples labeled yellow at the Lake Hawassa Watershed.

TDS plays a critical role in estimating the suitability of water bodies for both domestic and agricultural uses [97]. The overall mean value of TDS and EC in the study watershed was 598.7 mg/L and 1207.4 µS/cm, respectively (Table 7 and Figure 5a,b). The samples in the study watershed fell well below the prescribed values (<2000 mg/L) for TDS and (<3000 µS/cm) for EC recommended for human consumption and agricultural purposes [65,98]. However, samples collected from MS4, MS5 and MS15 were far above the recommended limits for human consumption and agricultural purposes. The mean value of TDS in this study for Lake Hawassa (455.6 mg/L) were greater than that of Lake Ziway (200 to 400 mg/L) conducted by Hengsdijk and Jansen [99].

The major source for TDS is due to livestock waste, landfills and dissolved minerals [100]. Electrical conductivity can be categorized low when EC ≤ 250 µS/cm (C1), medium when 250–750 µS/cm (C2), high when it ranges from 750–2250 µS/cm (C3) and very high when > 2250 µS/cm (C4) [100,101,102]. If applied for irrigation, high salt concentration (high EC) in water leads to formation of saline soil and a high sodium concentration leads to development of an alkaline soil.

#### 3.2.2. Sodium Adsorption Ratio (SAR) and Kelly Ratio (KR)

The appropriateness of water bodies for agricultural purpose was estimated by computing several parameters like salinity (EC), sodium absorption ratio (SAR), Kelly’s ratio (KR), soluble sodium percentage (SSP) and magnesium adsorption ratio (MAR) [100]. The above parameters were categorized based on the literature review [98,103].

The average EC in the study watershed ranged from 168.62 µS/cm (MS1) for Wesha River to 4257.4 µS/cm (MS5) for the Moha soft drink factory. The intake of water by plants decreases with increasing TDS or electrical conductivity value of water. Hence, the maximum yield reduction of crops occurred when the EC in agricultural water exceeds 3000 µS/cm [98,104]. SAR values for each water sample were calculated by using the following Equation [101].

(20) $$\mathrm{SAR}=\frac{{\mathrm{Na}}^{+}}{\sqrt{\left(\frac{{\mathrm{Ca}}^{+2}+{\mathrm{Mg}}^{+2}}{2}\right)}},$$

All the ions are expressed in meq/L.

Water for agriculture can be considered excellent if SAR < 10, good when SAR is 10–18, fair if SAR is 18–26, and SAR values above 26 are unsuitable for agricultural use [100,101].

**Figure 6:**
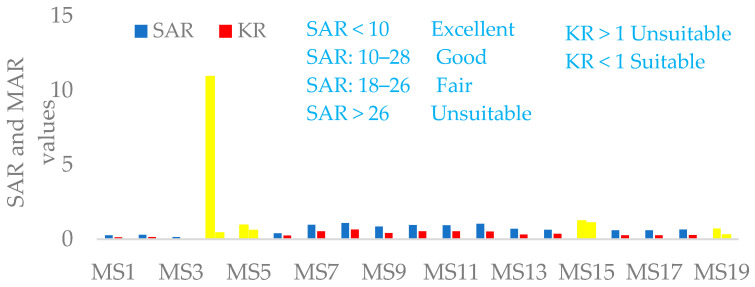
Sodium adsorption ratio (SAR) (meq/L) and Kelly’s ratio (KR, meq/L) in the water and wastewater samples (n = 19) collected over 19 monitoring stations with wastewater samples labeled yellow at the Lake Hawassa Watershed.

In the present study, all the monitoring stations fell in the excellent class, i.e., the SAR values < 10 except for one sampling site (MS4) collected from BGI which fell under the good category. Samples categorized under excellent and good could be used for agriculture with respect to SAR values (Table 7 and Figure 6).

The exchangeable sodium ratio higher than 1 is an indication of an excess level of sodium in waters in comparison to calcium and magnesium. Thus, waters with a KR ratio more than one are unsuitable for irrigation, while those with a ratio less than one are suitable. In the Lake Hawassa watershed, KR ranged from 0.05 (MS3) to 1.08 (MS5) indicating that nearly all sampled water values are less than the prescribed limit and suitable for irrigation [105].

(21) $$\;\mathrm{KR}=\frac{{\mathrm{Na}}^{+}}{{\mathrm{Mg}}^{+2}+{\mathrm{Ca}}^{+2}}$$

All the ions are expressed in meq/L.

#### 3.2.3. Soluble Sodium Percentage (SSP) and Magnesium Adsorption Ratio (MAR)

Wilcox [106] has suggested a classification system for ranking agricultural water use depending on SSP and estimated using the formula below.

(22) $$\mathrm{SSP}=\frac{{\mathrm{Na}}^{+}}{{\mathrm{Na}}^{+}+{\mathrm{Ca}}^{+2}+{\mathrm{Mg}}^{+2}}*100,$$

All the ions are expressed in meq/L.

**Figure 7:**
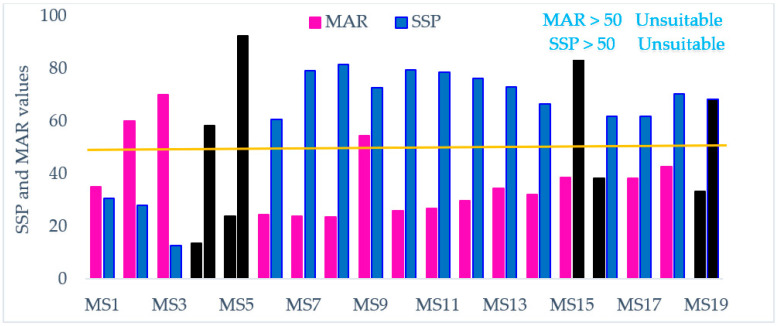
Soluble sodium percentage (SSP) and magnesium adsorption ratio (MAR) values (%) in the water and wastewater samples (n = 19) collected over 19 monitoring stations with wastewater samples labeled black at the Lake Hawassa Watershed.

SSP values above 50% mean the sampled water is not suitable for agricultural use and values lower than 50% indicate good quality of water [106]. The values of SSP in the watershed under investigation were far above the recommended limit (>50%) by Wilcox except samples collected from monitoring stations MS1(30.79%), MS2 (28.14%) and for MS3 (12.86%) (Table 7 and Figure 7).

Extreme concentrations of Mg^+2^ in agricultural water might be injurious to crops owing to the reduced availability of K^+^ in soils where magnesium concentrations are elevated. In Lake Hawassa Watershed, all samples but three were found to fall under the “suitable” class for MAR. Samples taken from the upper catchment (Wesha, Hallo and Wedessa rivers) and one sample taken from Lake Hawassa near Lewi resort deviates from the permissible level (MAR > 50%) [107].

(23) $$\mathrm{MAR}=\frac{{\mathrm{Mg}}^{+2}}{{\mathrm{Ca}}^{+2}+{\mathrm{Mg}}^{+2}}*100,$$

All the ions are expressed in meq/L

### 3.3. Determination of WQI and Status of Lake Hawassa Watershed

#### 3.3.1. Weighted Arithmetic Water Quality Index (WA WQI)

**Figure 8:**
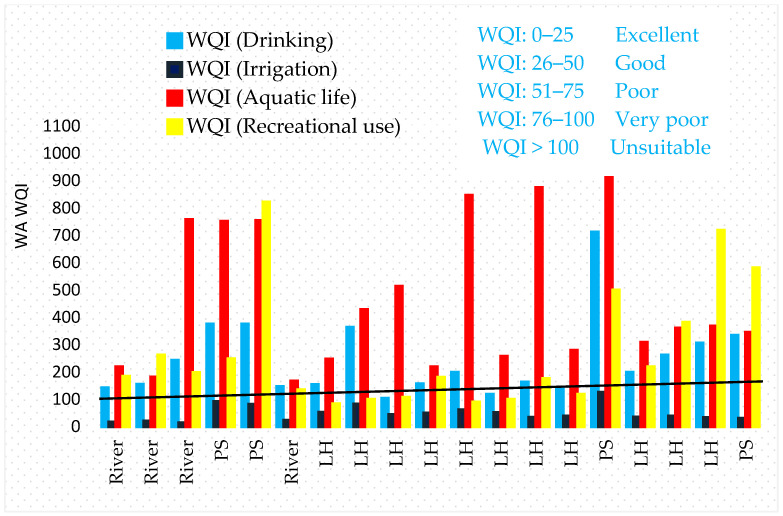
Weighted arithmetic water quality index (WA WQI) for drinking, irrigation, recreation and aquatic life in samples collected from rivers, PS (point source) and LH (Lake Hawassa) of the water and wastewater samples (n = 19) collected over 19 monitoring stations at the Lake Hawassa Watershed.

**Figure 9:**
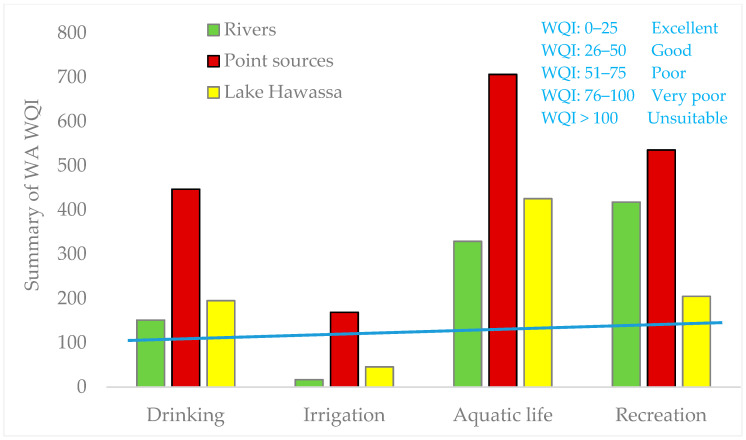
Summary of results for weighted arithmetic water quality index for drinking, irrigation, recreation and aquatic life for rivers, Point source and Lake Hawassa.

WA WQI and CCME WQI were used to integrate diverse parameters and their dimensions into a single score. The upshots of the physicochemical parameters of water for Lake Hawassa Watershed in 19 monitoring stations are presented in (Table 7, Figure 8 and Figure 9) and water quality status for each monitoring stations evaluated for drinking, irrigation, aquatic life and recreational purposes using WA WQI’s and ranked based on Table 3.

This water quality rating undeniably showed that WA WQI for the drinking use for rivers ranged from 81 (MS1) to 186 (MS3) and for Lake Hawassa it ranged from 72 (MS1) to 289 (MS3) and, therefore, can be categorized as unsuitable for drinking purposes (Figure 8).

WQI for irrigation for rivers ranged from 11.4 (MS3) to 20.8 (MS6), and for Lake Hawassa it ranged from 29 (MS13) to 66 (MS14). Amongst monitoring stations, samples analyzed from all rivers were categorized as excellent and the analyzed samples from Lake Hawassa were categorized under good for irrigation purposes.

Additionally, WA WQI was also computed to evaluate the suitability of the studied watershed for aquatic life and recreational purposes. It was observed that the computed WQI for rivers ranged from 159 (MS2) and 168 (MS1) and for Lake Hawassa it ranged from 210 (MS7) and 860 (MS13) for the aquatic life and, therefore, can be categorized as unsuitable. The water bodies in the studied watershed were also very poor and unsuitable for recreational purposes as the WA WQI was above 80 in all monitored water sources.

To compare monitoring stations, we divided them in to three categories by taking into consideration the upper catchment, the middle catchment and Lake Hawassa. WA WQI was computed separately for rivers in the upper catchment (MS1–MS3 and MS6), Industrial effluents and the Referral Hospital (MS4, MS5, MS15 and MS19) and Lake Hawassa (MS7–MS18) to pinpoint where the problem precisely lies.

The findings revealed that the water quality of rivers in the upper catchment namely Wesha, Hallo, Wedessa and Tikur-Wuha (middle part) and Lake Hawassa was generally unsuitable for drinking, aquatic life and recreational purposes. However, the rivers possessed excellent water quality and Lake Hawassa water quality was good for irrigation purposes (Figure 9).

**Figure 10:**
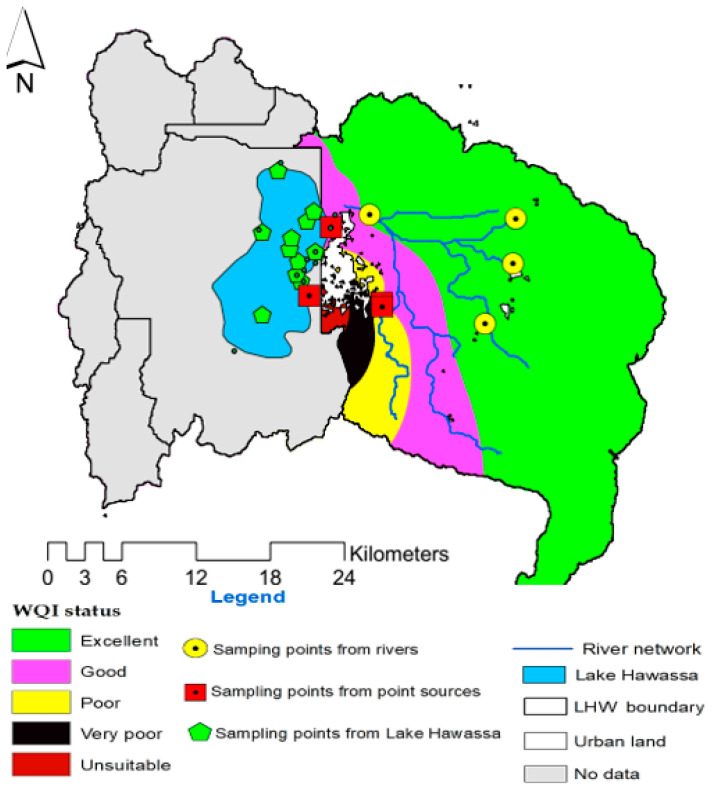
Topo to raster interpolation for estimation of Irrigation water suitability using WA WQI in the water and wastewater samples (n = 19) collected over 19 monitoring stations at the Lake Hawassa Watershed.

The topo to raster interpolation is among areal interpolation techniques that depict the spatial distribution of parameters. The interpolation result of appropriateness of water for agricultural use in Lake Hawassa Watershed revealed the irrigation water quality of the rivers on the uppermost part of the catchment is in the excellent and good classes that are in agreement with WA WQI and CCME WQI. The irrigation water quality of the watershed was reduced towards the industrial sites. The water quality deterioration is because the two point sources (Moha soft drinks factory and BGI) releasing their effluents into the adjoining rivers. Additionally, the result also showed unsuitability of water quality for irrigation in some points of the watershed due to samples taken from Referral Hospital, BGI, Moha soft factory showing the point sources were not in a good condition. Generally, this interpolation result also revealed the city of Hawassa takes the lion’s share in Lake Hawassa pollution followed by agricultural land use contribution. Here, the topo to raster interpolation was not applied for the western part of the Lake Hawassa Watershed, as there are no perennial streams feeding the lake (Figure 10).

**Figure 11:**
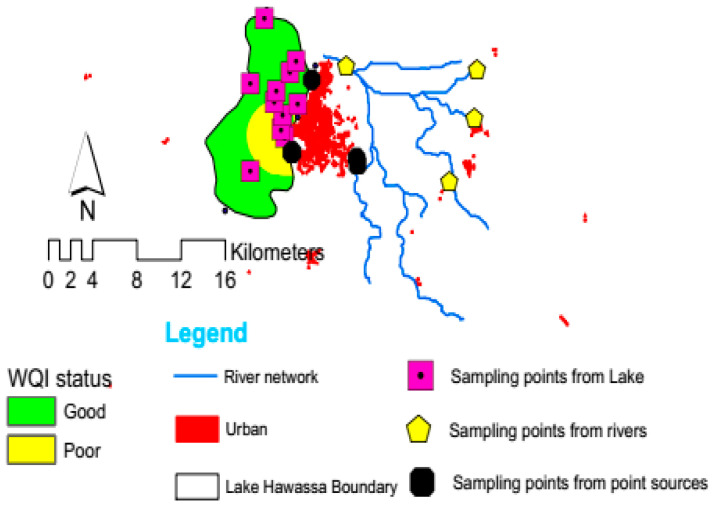
Topo to raster interpolation for estimation of Irrigation water suitability using WA WQI in the water samples (n = 11) collected over 11 monitoring stations at the Lake Hawassa.

Furthermore, the topo to raster interpolation of Lake Hawassa was conducted separately and the result showed that nearly all the sampling points are in the category of good quality for irrigation except one of the sampling points, the result of which was found to be poor for the sample taken from MS8 (Gudumale). This is because there was a small fish market near the site and it is nearer to the recreational facility that was serving for marriage ceremonies and for various events. Additionally, the site is near to the city of Hawassa, and as a result the urban runoff makes its way to the lake via this site. Additionally, this site is near to referral hospital and the service rendering facility. Due to this, the irrigation water quality is poor at this particular site. Generally, it was possible to conclude that the overall water quality of Lake Hawassa was in a good class for irrigation purposes, which is in agreement with WA WQI (Figure 11).

The findings are contrary to the previous studies conducted on the eastern catchment by Teshome [55] who found that higher values for WA WQI indicate the water quality of rivers and lake were unsuitable for all purposes. Also, the study conducted by Zemede et al. [10] on Lake Hawassa whose findings demonstrated Lake Hawassa is unfit for all purposes. Therefore, the cumulative result of WA WQI for drinking, aquatic life and recreational uses showed that the environmental situation has become worse in the last few decades, Hence, Lake Hawassa watershed has been polluted and frequent monitoring of the watershed is necessary for proper management.

A number of parameters affect the suitability of water for drinking, aquatic life and recreational purposes. Hence, dedicated efforts should be exerted to mitigate their release in to the rivers and Lake. Pollution prevention and control measures should be pursued as a matter of urgency by the pertinent figures.

#### 3.3.2. Canadian Council of Ministries of the Environment Water Quality Index (CCME WQI)

In this study, CCME WQI was employed using 21 water quality parameters (pH, EC, TDS, Turbidity. NH_3_, NH_3_-N, NO_2_^−^, NO_3_^−^, NO_3_-N, DO, BOD, COD, Mg^+2^, Ca^+2^, Na^+^, K^+^, Temperature, SAR, KR, MAR, SSP and SD) from May 2021 to December 2021 to evaluate the suitability of Lake Hawassa Watershed for drinking, irrigation uses, recreational use and aquatic life.

CCME WQI gives parameter values mathematically to confirm each parameter contributes sufficiently in the ultimate quality index. The values of each analyzed parameter were compared to the limits set by various international and national standards like WHO, CCME, EPA, FAO and other recommendations for those envisioned purposes. After the CCME WQI value was calculated in relation to monitoring site, month and the watershed, water quality was ranked as per the CCME WQI category (Table 4).

**Figure 12:**
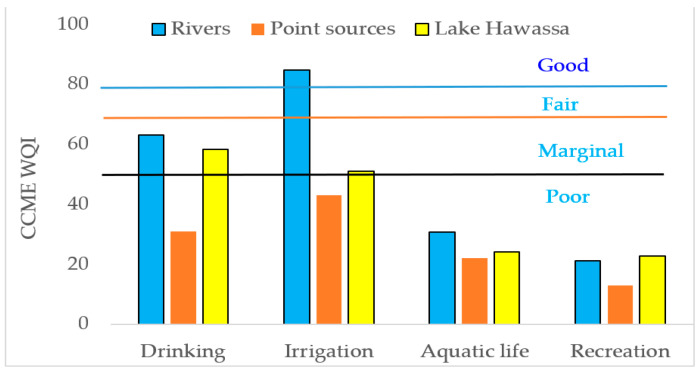
Summary of results for CCME WQI for drinking, irrigation, recreation and aquatic life for rivers, Point source and Lake Hawassa.

The results of the physicochemical parameters of water for Lake Hawassa Watershed in months and parameters were presented in Table 8 and Figure 12. The water quality status of four rivers in the upper and middle catchments (Wesha, Hallo, Wedessa and Tikur-Wuha) and four point sources in the middle of the catchments (BGI, Moha soft drinks factory, Hawassa Industrial park and Referral Hospital) and Lake Hawassa and its suitability evaluated for drinking, irrigation, aquatic life and recreational purpose using CCME WQI’s. The CCME WQI assessment result for rivers and Lake Hawassa showed that they are unsuitable for monitored parameters for marine life and recreation and marginal for drinking and irrigation purposes. However, the CCME WQI results for rivers fell in the good quality class, whereas the lake water assessment showed the CCME WQI result is marginal for irrigation purposes. This might be due to CCME WQI is sensitive to failed parameters and values change gradually between the lower classes.

The CCME WQI calculation for the envisioned water uses on Lake Hawassa Watershed was estimated for rivers, Lake Hawassa and point sources solely. The investigation depicted the four rivers in the upper and middle catchments (Wesha, Hallo, Wedessa and Tikur-Wuha) falls under good category for agricultural purposes (CCME WQI 85), marginal and poor for domestic (CCME WQI 64), marine life (CCME WQI 39) and recreational purposes (CCME WQI 21) respectively (Figure 12).

CCME WQI were computed for four point sources in the middle of the catchments (BGI, Moha soft drinks factory, Hawassa Industrial park and referral Hospital) separately. The findings exhibited poor or (impaired/threatened) water quality status for drinking (CCME WQI 39), aquatic life (CCME WQI 25), recreational uses (CCME WQI 13) and irrigation having the index value of (CCME WQI 43).

As far as Lake Hawassa is concerned, the water quality suitability was also evaluated for drinking and irrigation purposes and fell under the marginal category (CCME WQI 61, 51 respectively) and was poor for aquatic life and recreational purposes (CCME WQI 29, 23 respectively). The results of this study were comparable to the previous studies conducted by Zemede et al. [10] whose assessment result was marginal for all purposes and Worako [108] whose assessment showed the lake water is marginal for drinking and recreation but fair for irrigation and aquatic life on Lake Hawassa (Table 9).

The findings prove that there was a higher level of contamination for a broad range of substances. The point sources were the stronger polluter of the lake and, in particular, the Moha soft drinks factory and Referral Hospital were releasing extremely high values of some pollutants to the receiving environments.

The water bodies in Lake Hawassa Watershed was found to be impaired or unsuitable for the best uses of water especially for human consumption, recreational activities and aquatic life and in some cases for irrigation use as well due to agricultural run-off, effluents from surrounding industrial sectors.

### 3.4. Estimation of the Trophic Status of Lake Hawassa

#### 3.4.1. Analysis of Trophic State Variables

**Figure 13:**
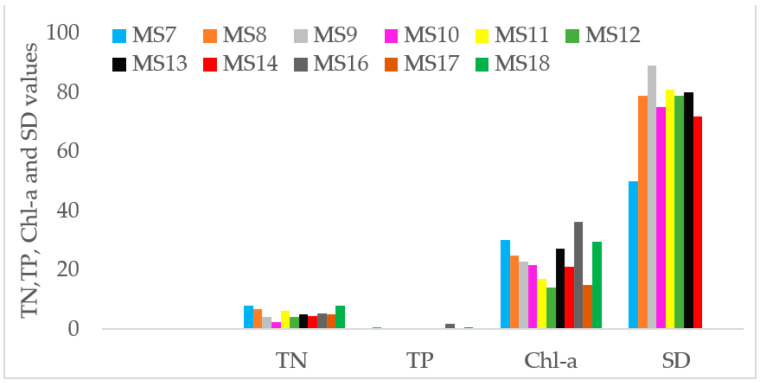
TN and TP concentrations (mg/L), SD depth (cm) and Chl-a concentrations (µg/L) in the water samples (n = 11) collected over 11 monitoring stations at Lake Hawassa.

Total phosphorus (TP), total nitrogen (TN), chlorophyll-a (Chl-a) and Secchi depth (SD) for the analysis of the trophic status of Lake Hawassa are shown in Figure 13.

##### Total Phosphorus (TP) and Total Nitrogen (TN)

TP ranged from 1 µg/L to 1843.75 µg/L with an average concentration of 317.5 µg/L TN ranged from 2.23 mg/L (MS10) to 7.87 mg/L (MS18) with mean concentration of 5.33 mg/L. The Kruskal–Wallis test result showed that TP and TN concentrations among sampling sites were not statistically different (*p* > 0.05). Gurung et al. [63] and Lau et al. [61] classified the trophic status based on the level of phosphorous and nitrogen. The lake is labelled as oligotrophic when TN (0.65–1.2) mg/L, TP (0.03–0.1) mg/L, mesotrophic if TN (0.35–0.65) mg/L, TP (0.01–0.03) mg/L, eutrophic when TN (0.65–1.2) mg/L and TP (0.03–0.1) mg/L and hypertrophic if TN > 1.2 mg/L and TP > 0.1 mg/L (Table 6).

Hence, Lake Hawassa is categorized as hypertrophic having mean TP and TN value of 320 µg/L and 5.33 mg/L, respectively. Phosphorus concentration greater than 300 µg/L [109] shows the impairment of the lake water by anthropogenic factors. The findings are comparable to the previous studies conducted on Lake Hawassa by Worako [6] and lower than the findings of Tibebe et al. [76] conducted on Zeway Lake.

##### Total Nitrogen to Total Phosphorus (TN:TP) Ratio

The TN:TP ratio in lakes and reservoirs is a key element as it gives an idea of which of these nutrients are either in excess or limiting to growth, and it was used to estimate the nutrient limitation in the lake. According to Smith [110] blue-green algae (cyanobacteria) had a capacity to dominate in the lake section when the TN:TP ratio was less than 29 and it tends to be rare in the lake when TN:TP > 29.

On the other hand, Fisher et al. [111] used more conservative ratio of the TN:TP and the ratio > 20 designated as phosphorus limitation and nitrogen limitation when the ratio is <10, while TN:TP ratio 10 to 16 demonstrates either phosphorus or nitrogen (or both) are limiting for growth. The estimated ratio for Lake Hawassa was 31.1 which is higher than 20 and 30 in the lake under investigation revealing cyanobacteria dominance in the lake section is rare. The TN:TP ratio > 20 in Lake Hawassa indicated that phytoplankton growth in the lake might be phosphorous deficient. Studies conducted on some Rift Valley lakes, namely Lakes Ziway and Hawasa, by Tilahun and Ahlgren [29], and Lake Zeway by Tibebe et al. [76] revealed that the lakes were found to be phosphorus limiting having a TN:TP ratio higher than 20.

##### Secchi Depth (SD) Chlorophyll a (Chl-a)

The average SD in Lake Lake Hawassa ranged between 0.5 m to 0.89 m with a mean value of 0.76 m. Smith et al. [112] categorizes the status of the lake based on the Secchi depth and the lake is designated as hypertrophic if SD (m) < 1, mesotrophic when SD (1–2), eutrophic if SD (2–4) and oligotrophic if SD > 4. Hence, Lake Hawassa is categorized as hypertrophic since the mean SD is 0.76 m. Chlorophyll a ranged between 14 µg/L and 30 µg/L with a mean of 23.6 µg/L in this study (Figure 12). A study conducted by Fetahi and Mengistou [113] and Tilahun and Ahlgren [29] showed the phytoplankton biomass measured on Lake Hawassa was 10.4 to 25.2 and 13 to 26, respectively, and the results are comparable to the present study.

#### 3.4.2. Evaluation of the Trophic Status Using Carlson Trophic State Index (TSI) Model

**Figure 14:**
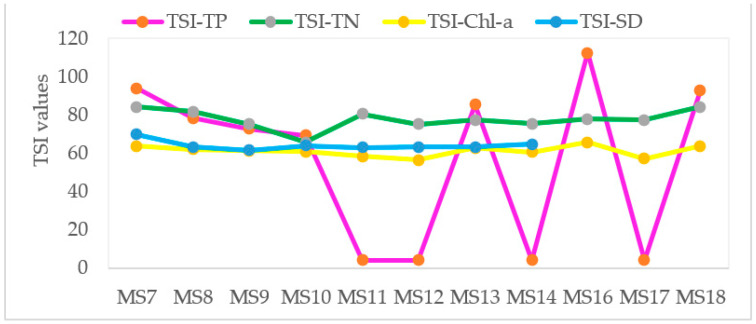
TSI-TN concentrations (mg/L), TSI-TP and TSI-Chl-a concentrations (µg/L) and TSI-SD depth (cm) of trophic variables in the water samples (n = 11) collected over 11 monitoring stations at Lake Hawassa.

The average TSI-TP, TSI-TN, TSI-Chl-a and TSI-SD value were 56.6, 77.8, 61.2 and 64.2, respectively. The trophic state is classified as oligotrophic (TSI < 40), mesotrophic (40 ≤ TSI < 50), eutrophic (50 ≤ TSI < 70) and hypertrophic (TSI ≥ 70) according the TSI values [22,60]. TSI values of TN of Lake Hawassa was above the eutrophic threshold. The whole average TSI of Lake Hawassa was 65.4 and, hence, the overall class of the lake falls under eutrophic state (Table 5 and Figure 14). The findings of this study were different from that of Zemede et al. [10] whose finding was hypertrophic as the assessment result depended only on the Secchi depth and also Worako [6] who found an average TSI of 72.6 (hypereutrophic) for Lake Hawassa. Eutrophication causes the impairment of activities, discomfort and visual unpleasantness that hamper the recreational use of water severely [85].

## 4. Conclusions

WQIs connote analysis of a variety of parameters into a sole value that offers the chance to evaluate existing water quality situations by classifying water bodies into definite classes. Likewise, in WQIs a communal summary for reference is provided for ranking different water bodies and identifying variations in quality conditions. Despite the various uses, WQI might not convey satisfactory information about the existing water quality statuses of water bodies. Therefore, the water users and water authorities may adopt the WQIs with slight modifications to conform to local situations.

This investigation made use of two different WQI indices to evaluate the water quality status of Lake Hawassa Watershed in order to obtain comparative performance of the different approaches. In addition, Carlson’s TSI, were used to obtain a comprehensive visualization of the quality of Lake Hawassa. The water quality of the watershed was broadly classified into unsuitable to excellent based on the envisioned usage and sampling locations. The findings of the water quality index of rivers and lakes showed, they were unsuitable for drinking, marine life and recreational purposes. In particular, in WA WQI classifications rivers fall in the excellent category and Lake Hawassa falls in the good category for irrigation purposes; while, the CCME WQI is a conservative approach whose range of values change gradually between the lower classes and rivers fall in the good category and Lake Hawassa falls in the marginal category for irrigation purposes. Hence, the discrepancy in the results of the two indices observed are imperative in order to consider measures that are more reliable. The topo to raster interpolation were carried out for both the watershed and Lake Hawassa separately and the results were in line with the findings of WA WQI and CCME WQI. To sum up, according to all indices, both lake and river water are unsuitable for drinking, marine life and recreational purposes. Even for irrigation purposes, the lake water is not suitable. Similarly, the overall category of Lake Hawassa falls under the eutrophic state and the lake is phosphorous-deficient. Similarly, the overall category of Lake Hawassa falls in the eutrophic state and the lake is phosphorous-deficient. These alarming assessments show the urgent need for pollution mitigation and control measures as a matter of urgency.

The findings publicize that the lake is suffering from various deficits, high nutrient concentrations, ammonia toxicity and oxygen depletion. The high COD and BOD values are partly due to direct emissions but also to the growth of organic matter in the lake water. Its degradation leads to reduced DO levels or even anaerobic conditions in deeper layers. The resulting threat to marine life is also endangering the fishery in the lake.

Also, the observed high nutrient concentrations and ammonia toxicity were attributed to TN, TP, NO_2_^−^, NO_3_^−^, TP, SRP and the un-ionized form of ammonia. These values emanated from the direct release from point sources that are the principal contributors and non-point sources such as agricultural land use (inorganic nitrogen and phosphorous fertilizers) and urban runoff during rainy season.

The findings of the study showed the environmental situation became worse in the last decade and Lake Hawassa watershed is known to be polluted. The dramatic worsening of the situation in Lake Hawassa Watershed was due to urbanization, usage of fertilizers in agricultural lands, effluents from industrial facilities, excessive usage of detergents in domestic and industrial facilities, soil erosion, increased sewage pollution, practice of open defecation and urban runoff. On top of that, there is insufficient sanitation in Lake Hawassa Watershed from diffused sources like sewage, animal waste pollution and the practice of open field defecation. The point sources have been known to take the leading role in contributing more pollutants to the river and Lake Hawassa followed by non-point sources from agricultural and urban runoff showing an ongoing pollution.

The monitored point sources indicate that the city of Hawassa and its numerous industrial discharges are key polluters, requiring a fast and consequent set-up of an efficient wastewater infrastructure, accompanied by a rigorous monitoring of large point sources (e.g., industry, hospitals and hotels). In spite of the various efforts, the recovery of Lake Hawassa may take long time as it is hydrologically closed. Therefore, to ensure safe drinking water supply, a central supply system according to WHO standards also for the fringe inhabitants still using lake water is imperative. Introducing the riparian buffer zones of vegetation and grasses can support the direct pollution alleviation measures and is helpful for reducing the dispersed pollution coming from the population mostly using latrines. Additionally, integrating aeration systems like pumping atmospheric air into the bottom of the lake using solar energy panels or diffusers are effective mitigation measures that will improve the water quality of the lake. In parallel, implementation and efficiency control of measures requires coordinated environmental monitoring with dedicated development targets.

## Figures and Tables

**Table 1:** Monitoring stations in Lake Hawassa Watershed.

Code	Monitoring Sites	Latitude (Y)	Longitude (X)	Altitude (Z)
MS1	Wesha river	783,404	457,401	1746
MS2	Hallo river	779,736	457,149	1724
MS3	Wedessa river	774,914	454,915	1764
MS4	BGI effluent discharge site	776,594	446,537	1686
MS5	Moha soft drinks factory	776,274	446,603	1671
MS6	Tikur-Wuha river	783,685	445,564	1677
MS7	Amora-Gedel (Fish market)	778,279	439,983	1676
MS8	Amora-Gedel (Gudumale)	778,862	439,661	1672
MS9	Nearby Lewi resort	779,941	439,791	1683
MS10	Central part of lake (Towards FH)	780,752	441,161	1681
MS11	Fikerhayk(FH) Recreation center	780,917	439,074	1690
MS12	Center of the lake (towards HR)	781,802	439,253	1682
MS13	Nearby Haile resort	783,146	440,463	1685
MS14	Tikur-Wuha site	784,000	441,060	1675
MS15	Referral Hospital	777,088	440,668	1686
MS16	Ali-Girma site (opposite to HR)	787,245	438,164	1690
MS17	Sima site (opposite to mount tabor)	782,325	436,885	1686
MS18	Dore-Bafana Betemengist	775,606	436,876	1683
MS19	Hawassa Industrial Park	782,669	442,464	1690

The site codes indicated in Figure 1, FH designates Fikerhayk and HR designates Haile resort.

**Table 2:** Analytical methods and instruments used for analysis.

Parameters	Analytical Method and Instrument
pH, EC, TDS and Temperature	Portable multi-parameter analyzer, Zoto, Germany
Turbidity	Nephelometeric (Hack, model 2100A)
DO	Modified Winkler
BOD_5_	Manometric, BOD sensor
COD	Closed Reflux, Colorimetric
SRP and TP	Spectrophotometrically by molybdovandate, HACH, model DR3900
Secchi depth	Standard Secchi disk of 20 cm, Secchi disk, LaMotte 20 cmD, USA
NO_3_^−^	Photometric measurements, Wagtech Photometer 7100 at 520 nm wavelength
NO_2_- and TAN (NH_4_^+^-N + NH_3_-N)	Spectrophotometrically by salicylate, (Hach, model DR3900)
TN	Spectrophotometrically by TNT Persulfate digestion, (HACH, model DR3900)
Mg^+2^, Na^+^, K^+^ and Ca^+2^	Atomic Absorption Spectrophotometer, AAS, (Hach, model NOVAA400)

TAN designates Total Ammonium nitrogen.

**Table 3:** Water quality index (WQI) and water quality rating.

WQI	Water Quality Rating
0–25	Excellent
26–50	Good
51–75	Poor
76–100	Very poor
>100	Unsuitable

**Table 4:** Canadian Council of Ministries of the Environment Water Quality Index (CCME WQI) Water quality categorization.

WQI	Water Quality Status	Remark
95–100	Excellent	Water quality is protected with a virtual absence of threat or impairment; conditions very close to the natural or pristine conditions. These index value can be obtained if all measurements are within objectives virtually all of the time.
80–94	Good	Water quality is protected with only a minor degree of threat or impairment: conditions rarely depart from natural or desirable levels.
65–79	Fair	Water quality is usually protected but occasionally threatened or impaired; conditions sometimes depart from natural or desirable levels.
45–64	Marginal	Water quality is frequently threatened or impaired; conditions often depart from natural or desirable levels.
0–44	Poor	Water quality is almost always threatened or impaired; conditions usually depart from natural/desirable level.

**Table 5:** Range of the Carlson’s Trophic Status Index (TSI) values and classification of lakes.

TSI	Classification	Description
<40	Oligotrophic	Deep lakes still exhibit classical oligotrophy, but some shallower lakes become anoxic in the hypolimnion during the summer.
40 ≤ TSI < 50	Mesotrophic	Water moderately clear, but increasing pro ability of anoxic in hypolimnion during summer.
50 ≤ TSI < 70	Eutrophic	Dominance of blue-green algae, algal scum probable, extensive macrophyte problems.
TSI ≥ 70	Hypereutrophic	Algal scum, summer fish kills, few macrophytes, dominance of rough fish.

**Table 6:** Trophic classification of lakes based on total nitrogen and total phosphorous.

Trophic State	TN (mg/L)	TP (mg/L)
Oligotrophic	<0.35	<0.01
Mesotrophic	0.35 ≤ TN < 0.65	0.01 ≤ TP < 0.03
Eutrophic	0.65 ≤ TN < 1.2	0.03 ≤ TP < 0.1
Hypertrophic	TN > 1.2	TP > 0.1

**Table 7:** Descriptive statistics: mean and standard deviation (in bracket) of the physicochemical characteristics for 19 monitoring stations in Lake Hawassa Watershed for evaluation of WA WQI for drinking, irrigation, aquatic life and recreational purposes.

Parameters	S1/S2/S3/S4/S5	MS1	MS2	MS3	MS4	MS5	MS6	MS7	MS8	MS9	MS10	MS11	MS12	MS13	MS14	MS15	MS16	MS17	MS18	MS19
Turbidity	5 ^ac^ 50 ^d^	11.7 (3.7)	27.3 (18.3)	34.8 (8.7)	20.5 (14.2)	21.7 (10.1)	12.2 (0.2)	7.4 (2.5)	7.4 (0.4)	5.7 (1.3)	6.1 (0.5)	7.2 (0.1)	6 (0.8)	7.8 (1.7)	7.3 (3.4)	14 (1.7)	8.8 (0.2)	7.6 (0.1)	46.5 (5.9)	4.2 (0.6)
TDS	1000, 2000 ^ab^	84.3 (6.9)	83 (24)	79 (11.7)	1704 (183)	2129 (312)	224 (132)	391 (4)	484 (48.6)	417 (37)	458 (21)	464 (2.1)	464 (4.7)	412 (0.8)	247 (157)	1491 (199)	476 (9.2)	475 (5.1)	518 (61)	776 (409)
EC	1500, 3000 ^ab^	169 (14)	166 (48)	158 (23)	3768 (81)	4257 (623)	446 (266)	776 (16)	835 (23)	822 (104)	932 (33)	924 (62)	928 (10)	799 (11)	491 (316)	2984 (399)	882 (75)	908 (38)	1084 (69)	1614 (6.3)
pH	6.5–9 ^abcd^	7.6 (0.8)	8.1 (0.8)	8 (0.4)	7.6 (0.7)	9.1 (0.5)	7.5 (0.03)	8.9 (0.1)	9 (0.1)	8.7 (0.001)	8.7 (0.2)	8.7 (0.1)	8.6 (0.04)	8.5 (0.1)	7.4 (0.1)	8.1 (0.2)	8.5 (0.2)	8.6 (0.02)	8.6 (0.2)	8.3 (0.1)
NH_4_-N		2.33 (3.23)	1.07 (1.24)	1.0 (1.26)	6.18 (4.1)	5.09 (1.56)	0.28 (0.29)	1.87 (2.18)	7.35 (3.52)	0.83 (0.67)	0.13 (0.003)	4.1 (0.6)	1.72 (0.53)	0.66 (0.72)	1.85 (0.93)	16.81 (14.55)	3.56 (0.01)	3.1 (0.01)	0.65 (0.18)	0.12 (0.09)
NH_3_	1.5, 1.37 ^b^	0.14 (0.2)	0.19 (0.27)	0.1 (0.13)	0.43 (0.36)	8.9 (6.31)	0.01 (0.01)	1.23 (1.52)	4.47 (1.29)	0.29 (0.22)	0.04 (0.01)	1.34 (0.48)	0.46 (0.14)	0.14 (0.14)	0.41 (0.15)	2.25 (2.36)	0.75 (0.22)	0.95 (0.07)	0.23 (0.14)	0.02 (0.01)
NO_2_^−^	3 ^a^	0.03 (0.01)	0.08 (0.08)	0.08 (0.01)	0.02 (0.01)	0.13 (0.01)	0.06 (0.01)	0.03 (0.01)	0.03 (0.002)	0.14 (0.15)	0.03 (0.03)	0.02 (0.01)	0.02 (0.02)	0.04 (0.04)	0.02 (0.001)	0.03 (0.02)	0.02 (0.01)	0.08 (0.02)	0.04 (0.02)	0.08 (0.004)
NO_3_^−^	45, 1 ^ac^	2.9 (0.02)	1.6 (0.04)	3.1 (0.5)	12.2 (0.1)	2.3 (0.8)	3.0 (2.3)	3.6 (0.7)	3.7 (0.7)	11.2 (3.3)	3.1 (0.8)	17.4 (6.8)	4.2 (0.4)	19.4 (7.8)	4.5 (1.6)	11.5 (6)	4 (0.4)	4.4 (0.02)	4.2 (0.02)	3.8 (0.5)
SRP	5 ^b^	5.3 (1.9)	14.3 (4.7)	4.5 (4.8)	20.2 (1.8)	76.8 (47)	4.1 (2.3)	2.3 (0.2)	3.2 (1.1)	2.3 (0.8)	2.5 (0.04)	1.8 (0.2)	2 (0.3)	2.9 (0.2)	3.6 (0.2)	28 (9.8)	3.5 (0.5)	2.7 (0.1)	6.9 (1)	8 (2.1)
DO	5 ^cd^	5.4 (1.8)	4.8 (1.8)	5.1 (1)	1.8 (0.4)	0.9 (0.04)	4.8 (1.2)	4.9 (0.7)	5.2 (0.1)	4.1 (0.02)	4.5 (0.3)	3.4 (0.1)	4.6 (0.04)	3.6 (0.6)	3.1 (0.5)	1.5 (0.04)	4.5 (0.2)	4.3 (0.3)	4.5 (0.4)	4.4 (0.41)
BOD	5 ^acd^	10.9 (4.1)	14.2 (13.4)	41 (40)	48 (21.6)	218 (131)	4.6 (0.9)	8.1 (3.1)	9.2 (0.4)	11.9 (2.9)	40.2 (44.7)	7.4 (2.3)	10.9 (1.0)	35.6 (16.5)	16.1 (5.8)	56.4 (9.9)	19.3 (4.6)	45 (4.2)	56.4 (1.3)	104 (30.6)
COD	20 ^a^	129 (58)	101 (9)	186 (181)	215 (69)	589 (393)	35 (12.4)	178 (87.1)	136 (1.4)	52.4 (10.5)	193 (189)	90 (7.8)	55.4 (13.2)	171 (119)	140 (8.8)	252 (53.7)	64.4 (15.7)	150 14.1)	188 (4.2)	416 (5.7)
Mg^2+^	200 ^a^	10 (4.2)	16.9 (10)	84 (98)	12.2 (1.1)	5 (3)	4.3 (2)	5.4 (0.5)	4.1 (0.2)	14.1 (1.9)	5 (0.4)	5.2 (0.3)	7.5 (3.7)	11.2 (3.3)	5.7 (0.9)	10.6 (44)	2.9 (0.4)	12.3 (2.5)	14.7 (1.9)	14.4 (5.5)
Ca^2+^	100 ^a^	32.8 (18)	17.4 (12)	19 (20)	43.8 (9.3)	26.4 (16)	22.5 (5.4)	21.5 (7.4)	22 (3.2)	19.5 (3.7)	25 (9.3)	23.8 (4.1)	28.8 (4.1)	35 (8.3)	20.5 (5.2)	35.7 (2.8)	7.8 (1.4)	32.6 (1.7)	32.4 (2.3)	46.9 (9.7)
Na^+^	200 ^a^	28.4 (5.8)	22.6 (5.1)	22 (5.3)	429 (101)	895 (259)	83 (41)	204 (26)	217 (54)	189 (4.6)	217 (10.1)	199.4 (8.1)	218.2 (9.6)	249 (45)	110 (58)	316 (148)	182 (21)	143 (22)	232 (17)	261 (58)
K^+^	20 ^a^	6 (0.9)	7.3 (1.1)	5.7 (1.5)	18 (2.5)	18.2 (1.6)	7.9 (2.2)	19.2 (1.3)	21.1 (1.5)	20.4 (0.5)	19.6 (0.6)	19.1 (2.2)	23.9 (0.8)	18.5 (0.7)	12.1 (6.5)	94.6 (70.6)	15.8 (3.2)	15.7 (3.2)	17.8 (1.8)	21.7 (1.2)
Temperature	15–20 ^ac^	17.4 (2.5)	16.6 (1.56)	17.2 (1.2)	33.6 (0.37)	30 (1.4)	23.2 (1.94)	22.6 (0.26)	22.3 (0.68)	22.2 (0.79)	20.6 (0.91)	22.6 (0.83)	21.6 (1.5)	23.2 (1.1)	20 (1.06)	25.4 (2.12)	22.1 (0.93)	23.1 (1.6)	23.8 (1.05)	21.36 (0.16)
SAR	26 ^b^	0.25 (0.1)	0.3 (0.03)	0.14 (0.04)	10.9 (0.05)	0.93 (0.37)	0.4 (0.12)	0.97 (0.2)	1.1 (0.02)	0.85 (0.03)	0.95 (0.15)	0.93 (0.16)	1.02 (0.06)	0.7 (0.07)	0.62 (0.37)	3.62 (2.81)	1.22 (0.12)	0.59 (0.09)	0.65 (0.04)	0.7 (0.14)
KR	1 ^b^	0.13 (0.08)	0.14 (0.01)	0.05 (0.03)	0.24 (0.01)	0.6 (0.4)	0.23 (0.08)	0.57 (0.19)	0.64 (0.02)	0.4 (0.02)	0.53 (0.12)	0.52 (0.12)	0.5 (0.04)	0.3 (0.07)	0.37 (0.23)	1.6 (1.3)	1.08 (0.02)	0.26 (0.02)	0.27 (0.002)	0.28 (0.1)
MAR	50 ^b^	34.9 (3.5)	60.2 (30.5)	70.2 (39.7)	32 (6.7)	24 (0.25)	24.4 (13.2)	30.3 (5.5)	23.8 (3.6)	54.7 (8.1)	26 (8.7)	26.9 (4.53)	30 (13.3)	34.5 (1.3)	32.2 (8.8)	32.6 (10.9)	38.6 (1.26)	38.4 (3.67)	42.9 (1.42)	33.3 (4.1)
SSP	50 ^b^	30.8 (14.9)	28.1 (1.96)	12.9 (8.5)	78.3 (1.97)	92.6 (5.8)	60.9 (12.8)	79.2 (6.2)	81.7 (1.8)	72.7 (0.06)	79.4 (5)	78.5 (2.8)	76.2 (0.3)	73 (1.9)	66.7 (14.7)	82.1 (0.34)	89.4 (0.33)	62 (1.24)	70.3 (0.42)	68.5 (1.8)

All units are in mg/L except turbidity, EC, temperature, SAR, KR, MAR, SSP and pH which were expressed in NTU, µS/cm, °C, meq/L, % and non-dimensional, respectively. (a) Labels drinking use, (b) irrigation water use, (c) express water use for aquatic life and (d) designates recreational water use. S1 labels Standard values taken from World Health organization (WHO), S2 labels Standard values taken from Environmental protection Agency (EPA) of US or Ethiopia, S3 labels Standard values taken from Canadian Council of Ministries of the Environment (CCME), S4 labels Standard values taken from Food for Agricultural organization (FAO) and S5 labels standard values taken from Health Canada (HC).

**Table 8:** The physicochemical characteristics of four rivers (Wesha, Hallo, Wedessa and Tikur-Wuha) for evaluation of CCME WQI in Lake Hawassa Watershed for drinking, irrigation, aquatic life and recreational purposes.

Parameters	MAY Rivers	JUNE Rivers	JULY Rivers	AUG Rivers	SEP Rivers	OCT Rivers	NOV Rivers	DEC Rivers	S1/S2/S3/S4/S5
Turbidity	37.4	31.7	26.0	20.3	18.9	17.5	16.0	14.8	5 ^ac^ 50 ^d^
TDS	87.5	85.8	84.0	82.3	94.6	126.5	148.8	170.0	1000, 2000 ^ab^
EC	178	173	169	165	179	253	298	340	1500, 3000 ^ab^
pH	8.1	8.2	8.2	8.3	8.0	7.7	7.5	7.2	6.5–9 ^abcd^
NH_3_	0.4	0.33	0.17	0.02	0.04	0.003	0.002	0.001	1.5, 1.37 ^c^
NH_3_-N	0.33	0.27	0.14	0.02	0.03	0.002	0.001	0.001	5 ^b^
NO_2_^−^	0.13	0.10	0.08	0.05	0.05	0.04	0.04	0.04	3 ^a^
NO_3_^−^	2.0	2.2	2.4	2.6	2.6	2.9	3.0	3.1	45, 1 ^ac^
NO_3_-N	0.4	0.5	0.5	0.6	0.6	0.7	0.7	0.7	10 ^b^
DO	6.2	6.2	6.1	6.1	5.6	4.7	3.9	3.4	5 ^cd^
BOD	8.0	6.8	5.6	4.4	11.8	20.2	28.1	35.6	5 ^acd^
COD	126	104	83	61	85	109	131	162	20 ^a^
Mg^2+^	7.6	7.2	6.8	6.4	22.4	38.8	54.9	71.6	200 ^a^
Ca^2+^	39.5	36.3	33.1	30.0	25.1	18.5	12.4	8.4	100 ^a^
Na^+^	30.2	28.6	27.0	25.3	33.9	41.2	49.2	56.8	200 ^a^
K^+^	5.5	5.6	5.6	5.6	6.3	7.1	7.8	8.4	20 ^a^
Temperature	20.6	20.3	20.0	19.7	19.1	18.1	17.3	16.7	15–20 ^ac^
SAR	1.2	1.2	1.2	1.2	1.5	1.8	2.2	2.5	26 ^b^
KR	0.6	0.6	0.6	0.7	0.8	0.9	1.2	1.4	1 ^b^
MAR	26.0	24.4	23.4	23.4	49.1	59.6	65.3	69.2	50 ^b^
SSP	35.6	36.1	37.4	40.1	35.8	37.4	40.6	41.8	50 ^b^

All units are in mg/L except turbidity, EC, temperature, SAR, KR, MAR, SSP and pH which were expressed in NTU, µS/cm, °C, meq/L, % and non-dimensional, respectively. (a) Labels drinking use, (b) irrigation water use, (c) express water use for aquatic life and (d) designates recreational water use. S1 labels Standard values taken from World Health organization (WHO), S2 labels Standard values taken from Environmental protection Agency (EPA of US or Ethiopia), S3 labels Standard values taken from Canadian Council of Ministries of the Environment (CCME), S4 labels Standard values taken from Food for Agricultural organization (FAO) and S5 labels standard values taken from Health Canada (HC).

**Table 9:** The physicochemical characteristics of Lake Hawassa for evaluation of CCME WQI for drinking, irrigation, aquatic life and recreational purposes.

Parameters	LH MAY	LH JUNE	LH JULY	LH AUG	LH SEPT	LH OCT	LH NOV	LH DEC	S1/S2/S3/S4/S5
Turbidity	9.8	10.9	10.9	11.4	11.1	10.7	10.4	10.0	5 ^ac^ 50 ^d^
TDS	466	427	412	402	419	436	453	471	1000, 2000 ^ab^
EC	941	852	821	778	811	844	876	911	1500, 3000 ^ab^
pH	9	8.7	8.5	8.3	8.4	8.5	8.5	8.6	6.5–9 ^abcd^
NH_3_	2.8	1.3	0.45	0.1	0.22	0.4	0.54	0.9	1.5, 1.37 ^c^
NH_3_-N	2.3	1	0.37	0.08	0.18	0.33	0.44	0.74	5 ^b^
NO_2_^−^	0.1	0.1	0.1	0.03	0.03	0.03	0.04	0.04	3 ^a^
NO_3_^−^	3.3	6.2	10.4	12.3	10.3	8.3	6.3	4.3	45, 1 ^ac^
NO_3_-N	0.7	1.4	2.3	2.8	2.3	1.9	1.4	1.0	10 ^b^
PO_4_^3−^	3.8	6.7	9.6	12.6	11.8	11.0	10.3	9.4	
DO	4.3	4.3	4.3	4.3	4.3	4.2	4.2	4.1	5 ^cd^
BOD	26.1	20.1	17.7	15.5	19.7	23.9	28.1	32.4	5 ^acd^
COD	122.0	106.3	113.2	118.6	126.8	134.9	143.0	151.2	20 ^a^
Mg^2+^	8.4	7.2	8.4	7.3	7.8	8.2	8.6	9.2	200 ^a^
Ca^2+^	33.3	28.1	24.9	19.8	21.2	22.5	23.8	25.2	100 ^a^
Na^+^	211.1	182.7	175.6	164.9	181.4	197.6	213.7	230.8	200 ^a^
K^+^	16.8	17.1	18.2	18.1	18.9	19.8	20.4	16.9	20 ^a^
Temperature	23.1	22.2	21.5	21.6	21.8	22.0	22.2	23.4	15–20 ^ac^
SAR	10.3	11.4	11.5	11.7	12.3	13.1	13.9	14.7	26 ^b^
KR	5.5	6.3	6.7	7.4	7.4	7.6	7.9	8.3	1 ^b^
MAR	33.1	40.8	45.3	51.4	51.2	50.8	50.2	49.8	50 ^b^
SSP	70.8	78.3	78.8	79.7	80.5	80.9	81.1	80.3	50 ^b^
SD	83.1	74.7	78.4	71.4	73.9	76.1	78.1	81.3	120 ^d^

All units are in mg/L except turbidity, EC, temperature, SAR, KR, MAR, SSP and pH which were expressed in NTU, µS/cm, °C, meq/L, % and non-dimensional, respectively. (a) Labels drinking use, (b) irrigation water use, (c) express water use for aquatic life and (d) designates recreational water use, LH designates Lake Hawassa. S1 labels Standard values taken from World Health organization (WHO), S2 labels Standard values taken from Environmental protection Agency (EPA of US or Ethiopia), S3 labels Standard values taken from Canadian Council of Ministries of the Environment (CCME), S4 labels Standard values taken from Food for Agricultural organization (FAO) and S5 labels standard values taken from Health Canada (HC).

## Data Availability

The data presented in this study are available on request from the first author. The data are not publicly available as it is experimental.
